# Plaque Progression and Rupture in Obstructive Coronary Artery Disease: A Review of Current Imaging Modalities

**DOI:** 10.31083/RCM48015

**Published:** 2026-04-24

**Authors:** Francesco Antonio Veneziano, Nino Cocco, Francesco Gentile, Francesco Chietera, Leonardo De Luca

**Affiliations:** ^1^Division of Cardiology, South Padova General Hospitals, 35043 Monselice, Italy; ^2^Operative Research Unit of Emodinamica, Fondazione Policlinico Universitario Campus Bio-Medico, 00128 Roma, Italy; ^3^Fondazione Toscana Gabriele Monasterio, Division of Cardiovascular Medicine, 56126 Pisa, Italy; ^4^Cardiology Unit, IRCCS University Hospital of Bologna, Policlinico S. Orsola, 40138 Bologna, Italy; ^5^Division of Cardiology, Fondazione IRCCS Policlinico San Matteo, 27100 Pavia, Italy

**Keywords:** coronary artery disease, atherosclerosis, plaque rupture, intravascular imaging, optical coherence tomography, coronary computed tomography angiography

## Abstract

Atherosclerotic plaque progression and rupture are the chief determinants of acute coronary syndromes and long-term outcomes in obstructive coronary artery disease (CAD). Residual risk persists despite intensive low-density lipoprotein-lowering and contemporary secondary prevention, because vascular inflammation and microstructural frailty often remain unresolved. At the bedside, the lesion that precipitates infarction is seldom the tightest but rather the most unstable. This review integrates the mechanistic chain, from endothelial dysfunction and retention/oxidation of apolipoprotein B lipoproteins to maladaptive innate and adaptive immunity, failed efferocytosis with necrotic core expansion, and biomechanical forces that thin and fatigue the fibrous cap, with their corresponding *in vivo* imaging phenotypes. Thus, this study aimed to examine how intravascular ultrasound (IVUS), optical coherence tomography (OCT), and near-infrared spectroscopy (NIRS), alongside coronary computed tomography angiography (CCTA), cardiac magnetic resonance (CMR), and positron emission tomography (PET), characterize these processes and enable longitudinal tracking of disease activity. Moreover, we briefly discuss emerging therapeutic implications of plaque imaging, focusing on how improved identification of vulnerable plaque features may inform risk stratification. Finally, we evaluate therapies that extend beyond lipid-lowering to modulate inflammatory and immune pathways, reinforce cap stability, and support a risk-adapted, trajectory-based pathway in which serial imaging and biomarkers guide treatment intensity. Together, these advances support a shift in clinical practice from stenosis-centered revascularization to imaging-guided, vulnerability-centered prevention.

## 1. Introduction

Coronary artery disease (CAD) remains the leading cause of morbidity and mortality worldwide, affecting more than 180 million people [1, 2]. Its clinical expression, including myocardial infarction and stroke, arises from a chronic and multifactorial atherosclerotic process in which lipid accumulation, inflammation, and structural destabilisation converge within the arterial wall. Over the past decade, there has been a progressive focus shift from the traditional “stenosis hypothesis”, related to the identification of flow-limiting stenoses, to a broader “plaque hypothesis”, which emphasizes biological vulnerability and mechanical instability [3].

Atherosclerosis is a chronic inflammatory disorder initiated by endothelial dysfunction, lipid retention and oxidation, and maladaptive immune responses at sites of disturbed shear stress [4]. Subendothelial lipid accumulation and oxidation trigger monocyte recruitment and macrophage activation, foam-cell formation, and release of pro-inflammatory cytokines. With persistent inflammation, a necrotic core develops beneath a thinning fibrous cap, rendering the lesion prone to rupture and thrombosis; this transition from stability to instability is driven by macrophage-mediated inflammation, protease activation, and impaired clearance of apoptotic cells. Recent advances have clarified the lipid–inflammation interplay: oxidised lipoproteins, hypoxia, and metabolic stress amplify macrophage activation and efferocytosis failure, establishing a self-perpetuating inflammatory loop within the plaque [5]. Immune activation, metabolic dysfunction, and clonal haematopoiesis further sustain vascular inflammation and residual cardiovascular risk despite optimal lipid-lowering therapy [6]. Inflammation therefore complements—not replaces—lipid-driven mechanisms, bridging metabolic and immune pathways in atherogenesis [5, 6].

Overall, this evidence reframes atherosclerosis as a dynamic, evolving, and systemic disease, and no longer a mere consequence of luminal obstruction. It is therefore crucial for clinicians to evaluate how biological, mechanical, and inflammatory processes intertwine to drive plaque progression and rupture, opening therapeutic avenues that shift the clinical focus from treating stenosis to stabilising vulnerability, setting the stage for the mechanistic, imaging, and translational sections that follow.

## 2. Pathobiology of Atherosclerotic Plaque Progression and Destabilisation

### 2.1 Cellular and Molecular Drivers of Plaque Progression

Atherosclerosis begins at sites of disturbed shear stress with endothelial dysfunction, increased permeability to apolipoprotein-B lipoproteins, and their subendothelial retention. Retained low-density lipoproteins undergo oxidative and enzymatic modification and trigger innate immune activation, monocyte recruitment, foam-cell formation, and a cytokine milieu sustained by NF-κB signalling and activation of the NLRP3 inflammasome [7].

Vascular smooth-muscle cells migrate and synthesize extracellular matrix, forming an initially stabilising fibrous cap; with persistent inflammatory and oxidative cues, they undergo apoptosis, matrix turnover accelerates, and phenotypic switching emerges towards macrophage-like, fibroblast-like, and osteochondrogenic states that contribute to plaque enlargement and calcification [8]. Endothelial cells exposed to disturbed flow activate mechanosensitive transcriptional programmes, including endothelial-to-mesenchymal transition, linking haemodynamics to regional vulnerability patterns [8, 9].

Impaired efferocytosis leads to secondary necrosis and expansion of the lipid core, while sustained cytokine signalling—such as interleukin-6 and tumour necrosis factor-α—maintains leukocyte influx. As microcalcification evolves into more confluent deposits, tissue stiffening further perturbs local shear and perpetuates endothelial injury. Viewed over time, progression reflects a multicellular, time-dependent remodelling process in which immune activation, vascular biomechanics, and metabolic stress interact; cumulative exposure to apolipoprotein-B lipoproteins and ageing govern the transition from subclinical lipid deposition to complex fibrotic and calcified lesions [10]. These changes establish the substrate upon which destabilisation is superimposed.

### 2.2 Mechanisms of Plaque Destabilization and Rupture

Plaque destabilization occurs when a lipid-rich but clinically silent lesion morphs into a biologically active substrate prone to rupture and thrombosis. It reflects converging axes—immune activation, extracellular-matrix degradation, programmed cell death, and haemodynamic stress—acting on an already remodelled wall. Persistent inflammation thins and weakens the fibrous cap by inducing smooth-muscle cell apoptosis and upregulating proteases that degrade collagen and elastin, culminating in structural failure [11].

Defective efferocytosis enlarges the necrotic core and creates an acellular, highly thrombogenic space enriched with tissue factor and cholesterol crystals. Hypoxia within this core activates hypoxia-inducible pathways, drives fragile neovascularisation, and favours intraplaque haemorrhage that accelerates growth of the lipid–necrotic zone [12].

The immune system both drives and executes this transition. Beyond classical cytokine signalling, trained immunity and clonal haematopoiesis generate hyper-responsive myeloid and T-cell populations that sustain inflammation and prime a prothrombotic milieu—often before any mechanical disruption is evident [13]. Two phenotypes dominate acute events. Rupture-prone plaques exhibit thin caps (<65 µm), large lipid pools, high macrophage density, and smooth-muscle depletion that co-localise with regions of elevated mechanical stress. Eroded plaques show endothelial denudation without cap fracture, fewer macrophages, abundant proteoglycans, and relative smooth-muscle preservation—indicating a distinct pathogenic route [14]. Haemodynamics modulate both. Low or oscillatory shear encourages inflammatory activation, while abrupt shear gradients concentrate circumferential stress at plaque shoulders and precipitate cap fatigue and fissuring [15]. At bifurcations, high-shear turbulence can detach endothelium and, together with leukocyte adhesion and neutrophil extracellular traps, promote thrombosis in the absence of rupture. Not all vulnerable-appearing plaques undergo disintegration: intensive lipid-lowering and anti-inflammatory therapies could positively modulate plaque, increasing the thickness of the fibrous cap and reducing the inflammatory substrate, while systemic susceptibility can keep lesions biologically “hot”. Consequently, the field has expanded from a vulnerable plaque to a vulnerable patient paradigm, integrating local characteristics with host biology. At the interface between structure and biology, the balance between immune activation and mechanical fatigue determines whether a plaque remains quiescent or triggers acute coronary occlusion [13, 16, 17].

## 3. Detection and Characterization of Vulnerable Plaque

### 3.1 Invasive Imaging: From Morphology to Biology

The transition from angiographic to intravascular imaging has profoundly reshaped our understanding of coronary atherosclerosis, transforming it from a disorder defined by luminal stenosis to a dynamic, multifactorial disease of the vessel wall. Invasive imaging techniques such as intravascular ultrasound (IVUS), optical coherence tomography (OCT), and near-infrared spectroscopy (NIRS) have emerged as complementary instruments that reveal the microstructural, compositional, and biological determinants of plaque vulnerability. These modalities collectively provide a mechanistic link between arterial remodeling, inflammatory activation, and biomechanical instability, allowing a direct visualization of coronary pathology that bridges clinical practice with molecular vascular biology.

IVUS represents the foundational technique for quantitative plaque evaluation. Its deep tissue penetration permits the assessment of global plaque burden, vessel remodeling, and the extent of compensatory enlargement—a phenomenon central to the concept of “outward remodeling” in high-risk lesions. The landmark PROSPECT and PROSPECT II trials [18, 19] established that lesions with a plaque burden ≥70%, minimal luminal area ≤4.0 mm^2^, or features consistent with thin-cap fibroatheroma (TCFA) confer a substantially elevated risk of subsequent major adverse cardiovascular events, even in the absence of flow-limiting stenosis.

Beyond geometry, modern implementations such as radiofrequency-IVUS and hybrid NIRS–IVUS have enabled tissue characterization and quantification of necrotic cores. However, the spatial resolution of IVUS (100–150 µm) remains insufficient to resolve the thin fibrous caps (<65 µm) that typify rupture-prone plaques [20, 21]. Despite this, IVUS remains indispensable for mapping the “macroarchitecture” of disease, offering insights into the spatial distribution of atherosclerosis, patterns of remodeling, and areas of low endothelial shear stress (ESS)—precursors of both progression and rupture.

OCT provides a nearly histological level of resolution (10–15 µm), revealing the fine structure of the fibrous cap, macrophage infiltration, and microchannels within the intima [22]. Unlike IVUS, which provides depth and volumetric context, OCT excels at detailing the microanatomy of vulnerability. Through its capacity to identify TCFAs, lipid pools, macrophage accumulations, and calcified nodules, OCT has become the gold standard for the *in vivo* identification of unstable plaques [23]. In patients with acute coronary syndromes, pooled OCT analyses show that the cumulative burden of traditional risk factors tracks with multiple hallmarks of vulnerability—including TCFA, macrophages, microvessels, and cholesterol crystals—with plaque rupture increasing and erosion decreasing as risk factors accumulate [24, 25]. The technique has refined the diagnostic differentiation between plaque rupture, erosion, and calcified nodule—the triad underlying most acute coronary syndromes. Moreover, longitudinal OCT studies have provided dynamic insights into therapy-induced remodeling: statins, PCSK9 inhibitors, and anti-inflammatory agents have been shown to induce measurable cap thickening and attenuation of macrophage signals, highlighting the method’s potential as a surrogate biomarker of therapeutic efficacy [26]. Still, OCT’s limited tissue penetration restricts visualization of deep lipid cores and positive remodeling, necessitating multimodal integration for full assessment.

NIRS contributes a biochemical dimension by detecting lipid-rich necrotic cores through their distinct absorption spectra. The lipid core burden index (LCBI), particularly the maxLCBI_4_mm, has emerged as a quantitative marker of lipid accumulation and inflammatory activity within plaques [27]. In PROSPECT II, the integration of NIRS-derived maxLCBI_4_mm ≥325 with IVUS-detected plaque burden ≥70% identified lesions with the highest likelihood of progression and future events [19, 28]. The availability of dual-modality catheters combining NIRS with IVUS or OCT now allows simultaneous assessment of plaque geometry, lipid distribution, and microstructural integrity within a single acquisition, providing a “biological fingerprint” of plaque composition. These hybrid systems have paved the way for an advanced, multidimensional model of coronary vulnerability in which lipid burden, cap integrity, and hemodynamic stress converge to define lesion risk. In parallel, a head-to-head comparison showed that coronary CT angiography had weak accuracy for detecting lipid-rich plaques versus NIRS–IVUS (≈58%), underestimated lumen and plaque volumes (e.g., total and percentage atheroma volume), and overestimated the lipid component, with potential implications for revascularisation planning [29]. In lesions considered for intervention, coronary CT angiography also underestimated reference vessel area and overestimated lesion length compared with NIRS–IVUS, reinforcing the role of intravascular imaging when precise compositional assessment and sizing are required.

Recent work has extended these modalities from static visualization to functional assessment of biomechanics. IVUS- and OCT-derived reconstructions can now estimate ESS and plaque structural stress (PSS), linking local mechanical forces with tissue remodelling. Low ESS fosters endothelial activation, lipid retention, and macrophage infiltration, whereas steep PSS gradients at plaque shoulders favour microfissuring and cap rupture [30].

When combined with NIRS-derived lipid content, stress mapping yields a three-dimensional view of how compositional and biomechanical vulnerabilities co-localise within the same lesion. In parallel, co-registration with computational fluid dynamics enables a form of “digital pathology” of the coronary tree—charting patterns of disease activity rather than lumen contours alone. In aggregate, high-resolution imaging and physics-based analysis are transforming the catheter laboratory into a functional observatory that stratifies risk beyond luminal obstruction and aligns intervention with the evolving biology of atherosclerosis—while hinting at an even finer layer of activity yet to be captured [24, 25, 31].

### 3.2 Non-Invasive Imaging: From Anatomy to Risk Prediction

Non-invasive imaging has moved beyond depicting luminal narrowing to a multidimensional appraisal of plaque biology. Coronary computed tomography angiography (CCTA), positron emission tomography (PET), and cardiac magnetic resonance (CMR) now quantify plaque geometry, composition, and the perivascular milieu, offering insight into inflammation, calcification, and remodelling before events occur.

CCTA remains the anchor modality for identifying high-risk features. Low attenuation (<30 Hounsfield units [HU]), positive remodelling (index >1.1), spotty calcification, and the napkin-ring sign reproducibly associate with culprit lesions and future events [32]; prospective cohorts (SCOT-HEART, PARADIGM) linked the presence and volume of low-attenuation plaque with myocardial infarction independently of stenosis [33, 34], and ROMICAT II showed incremental prognostic value in acute chest pain [35, 36]. The CRISP-CT framework introduced the perivascular fat attenuation index (FAI) as a quantitative marker of coronary inflammation that predicts cardiac and all-cause mortality beyond standard CT metrics [37]. Building on this, radiomic and machine-learning analysis extracts texture descriptors that capture heterogeneity, microcalcification patterns, and fat-attenuation variance; such models achieve high event-prediction accuracy and support data-driven “vulnerability scores” when integrated with clinical variables [38, 39]. Importantly, CCTA also clarifies risk at the clinical margins: in symptomatic adults ≤45 years, CAC is absent in the vast majority, yet CCTA still reveals non-calcified and even low-attenuation plaque in a meaningful minority—particularly among those with a family history of premature CAD—while ≥50% stenosis remains uncommon [40]. At the other end of the spectrum, in hospitalized patients with suspected or known CAD, the presence of any high-risk plaque (HRP) feature on CCTA identifies a group at substantially higher long-term mortality, whereas the absence of such features confers a prognosis comparable to those without CAD [41]. Consistently, sequential CCTA–PET work shows that CT-derived necrotic core volume independently relates to downstream perfusion (*p* = 0.029), and that adding composition improves prediction beyond stenosis or overall plaque burden, bridging morphology with function [42].

CMR adds a tissue-specific lens that extends assessment beyond the coronaries. High-intensity plaques on T1-weighted imaging—commonly expressed as a plaque-to-myocardium ratio (PMR) ≥1.4—associate with cap thinning, intraplaque haemorrhage, and subsequent events [43]. In CATCH T1-weighted MRI, a higher PMR independently predicts periprocedural myocardial injury and provides information complementary to lipid metrics from NIRS–IVUS [44]. Quantitative mapping (T1, T2, extracellular volume) can characterise fibrotic and lipid-rich/necrotic components and post-ischaemic remodelling, while advances in motion correction, accelerated acquisitions (including compressed sensing), and contrast-enhanced black-blood techniques have improved vessel wall visualisation and the assessment of oedema and inflammatory activity [45].

PET provides the functional counterpart to structural imaging. ^18^F-FDG highlights metabolically active macrophages, whereas fluorine-18 sodium fluoride (^18^F-NaF) localises microcalcification and extracellular-matrix turnover; focal NaF uptake in non-stenotic plaques predicts myocardial infarction, revealing subclinical disease activity [46]. Hybrid PET/CT and PET/MRI co-register tracer uptake with morphology (including FAI), and novel probes—^68^Ga-DOTATATE targeting somatostatin-receptor-2 on activated macrophages and ^18^F-Galacto-RGD binding αvβ3-integrins on neovessels—image macrophage activation and neoangiogenesis [47]. In parallel, CT-derived non-calcified plaque (NCP) volume and pericoronary adipose tissue attenuation (PCAT/FAI) co-segregate with OCT hallmarks of vulnerability and higher inflammatory tone, providing a structural–inflammatory scaffold onto which PET signals can be layered for finer biological resolution [48].

The trajectory is integrative. Multimodality pipelines combine CT’s anatomical coverage with PET’s molecular specificity or CMR’s tissue characterisation to build a biological atlas of coronary disease; computational fluid-dynamics modelling from CT geometries maps regions of low shear that co-localise with increased NaF uptake, uniting biomechanics and inflammation within a single framework [49]. Translationally, CCTA-derived plaque burden and FAI serve as surrogates of residual inflammatory risk to guide anti-inflammatory escalation or lipid-lowering intensification, while quantified NaF activity offers a pharmacodynamic endpoint for agents targeting calcification and oxidative stress [50].

### 3.3 Biological and Molecular Imaging: Capturing Plaque Activity Before the Event

The ultimate goal of cardiovascular imaging is increasingly to intercept changes in biological activity before it manifests clinically, based on the non-invasive markers described above.

Structural hallmarks remain necessary yet insufficient; instability originates in dynamic processes—cellular inflammation, oxidative stress, hypoxia, and matrix breakdown—that may antedate visible morphological change by weeks to months [51]. Within the catheter laboratory, invasive molecular imaging has reframed assessment from static inspection to dynamic surveillance.

OCT–NIRF co-localises cap microstructure with signals of inflammation, oxidative stress, and endothelial disruption, delineating macrophage-rich and fibrin-laden regions that explain why a thin cap is biologically fragile [52, 53]. Intravascular photoacoustic imaging (IVPA) merges optical excitation with ultrasonic detection to generate spectroscopic maps of lipid, cholesterol, and haemoglobin; hybrid IVPA–IVUS systems now achieve sub-75-µm axial resolution and can track oxygenation dynamics in real time, exposing pockets of metabolic instability aligned with known mechanisms of progression [54, 55].

Fluorescence lifetime imaging integrated with OCT adds biochemical contrast for collagen integrity and macrophage density at near-histological precision, turning the catheter lab into a living laboratory of atherogenesis with pharmacodynamic read-outs that extend beyond morphology. Aligned with an activity-before-event paradigm, OCT microstructural signatures (thin cap, broad lipid arc, macrophages) act as biological surrogates of instability: in the 5-year extension of CLIMA, the co-occurrence of all four prespecified features identified a small subgroup at markedly higher risk, and even any TCFA alone remained predictive of long-term adverse outcomes [51].

Non-invasively, hybrid PET–CT and PET–MRI move from anatomy to pathway-level biology.

Rather than reiterating FDG/NaF, the emphasis here is on specific tracers—^68^Ga-DOTATATE (somatostatin receptor-2 on activated macrophages) and ^18^F-Galacto-RGD (αvβ3-integrins on neovessels)—which image macrophage activation and neoangiogenesis and, when combined with MRI, align metabolism with perfusion, oedema, and tissue characterisation in a single sitting [56]. Complementary CT radiomics of the perivascular space provides a quantitative, operator-independent index of local cytokine activity that can be followed longitudinally [57].

Against this backdrop, CCTA supplies the whole-heart scaffold on to which these molecular signals can be mapped: it quantifies total plaque burden and phenotype, detects high-risk non-obstructive disease that functional tests miss, and—crucially—can be repeated to track progression and treatment response, enabling disease-centred, personalised prevention [3]. Technical advances—photon-counting detectors and deep-learning reconstructions—are further improving spatial/spectral fidelity at lower dose, while artificial intelligence (AI)-driven quantitative plaque analysis standardises measurement and facilitates fusion with PET and MRI read-outs [58].

The next step is integration across scales. Invasive read-outs (OCT–near-infrared fluorescence (NIRF), IVPA) define the plaque microenvironment, while PET–CT/PET–MRI and radiomic CCTA capture the systemic milieu. A concise biological activity index that merges fluorescence intensity, PET tracer uptake, and CT-derived inflammatory metrics treats vulnerability as a continuum rather than a binary state [37, 59].

Superimposing computational fluid dynamics on CT geometries then maps endothelial shear and PSS onto these molecular signals, shifting assessment from categorical to temporal, indicating not only where risk resides, but when it is most likely to manifest—thereby informing follow-up cadence and the timing of therapy escalation.

Quantitative thresholds commonly adopted across imaging modalities for plaque vulnerability assessment are summarized in Table 1 (Ref. [14, 19, 20, 21, 23, 27, 28, 32, 33, 34, 37, 46, 60, 61]).

**Table 1.** **Cross-modality quantitative anchors for a minimum reporting dataset (vulnerability assessment)**.

	Modality & metric	Working anchor (threshold)	Biological signal captured	Reference
1	OCT – minimum fibrous-cap thickness	<65 µm	Cap fragility/TCFA (collagen & SMC depletion)	[14, 20, 21, 23, 60]
2	NIRS – maxLCBI_4_mm	≥325	Lipid-rich necrotic core/macrophage activity	[19, 27, 28]
3	CCTA – low-attenuation plaque & burden	Low attenuation <30 HU; staged PAV	Necrotic-core surrogates and overall plaque burden linked to ischaemia/events	[32, 33, 34, 61]
4	CCTA – perivascular FAI	≈−70 HU (less negative = more inflamed)	Peri-coronary inflammation (residual risk)	[37]
5	PET – focal ^18^F-NaF uptake	Presence of focal uptake	Active microcalcification/matrix turnover predictive of events	[46]

**Acronym key**. OCT, optical coherence tomography; NIRS, near-infrared spectroscopy; LCBI, lipid core burden index; CCTA, coronary computed tomography angiography; PAV, percent atheroma volume; FAI, fat attenuation index; PET, positron emission tomography; ^18^F-NaF, fluorine-18 sodium fluoride; TCFA, thin-cap fibroatheroma; HU, Hounsfield units.

## 4. Translating Vulnerability Into Predictive Diagnosis and Clinical Integration

### 4.1 From Imaging Markers to Quantitative Standards

The foregoing sections establish that distinct technologies often describe the same biology; what is missing is a shared quantitative language that allows results to be compared, pooled, and tracked over time. A pragmatic core set of cross-modality anchors already exists: cap thickness <65 µm on OCT; maxLCBI_4_mm ≥325 on NIRS; staged percent atheroma volume (PAV) and low-attenuation plaque on CCTA; perivascular FAI close to –70 HU on CCTA; and focal ^18^F-NaF uptake on PET. These thresholds arise from invasive co-registration showing that OCT-defined TCFA co-localises with positive remodelling and lipid-rich substrate on NIRS–IVUS with high diagnostic performance [60]. In parallel, quantitative CCTA has moved assessment from lumenography to biology, with rising plaque burden and low-attenuation volume mapping to stepwise increases in ischaemia and events [61]. Meta-analytic heterogeneity persists when centres apply different cut-offs or segmentation rules, underscoring the need for calibration and a minimum reporting dataset that travels with the images as well as the numbers [62].

On that foundation, a Biological Activity Index can fuse morphology (cap/burden), lipid–inflammation (LCBI, FAI, NaF), and, where available, lesion biomechanics into a single, interoperable score—reported per lesion and per patient—to complement clinical risk and guide treatment intensity. It is crucial that the index be serial by design: the same metrics acquired with the same protocols allow for objective and reliable measurement of change, rather than being inferred. Coronary lesions have time-dependent characteristics, alternating cycles of activation and quiescence over the course of months. The prognostic outcome of a lesion depends not only on how it is assessed from the baseline snapshot, but rather on the direction and speed of change, which have prognostic weight [63].

After infarction, serial IVUS shows that non-culprit lesions can drift toward a higher-risk phenotype, with TCFA becoming more frequent, necrotic core expanding, and only modest changes in lumen area—indicating that adverse biology may worsen before stenosis visibly does [64, 65]. Moreover, vulnerability is not confined to tight lesions: across angiographic severities, the absolute number of TCFAs is greatest in non-severe segments, even though relative prevalence, cap thinning, plaque burden, and positive remodelling intensify as stenosis increases [66, 67]. Together, these observations support a trajectory-aware reading of follow-up imaging—flagging lesions whose microstructural risk is rising despite limited anatomical progression and prioritising earlier reassessment or treatment intensification when vulnerability signals accumulate [68].

Where available, computational shear and structural-stress maps can be overlaid on CT geometries to identify segments where mechanical fatigue and inflammatory activity co-localise, aligning surveillance and intervention with the window in which risk is most likely to declare itself [69]. Finally, plaque vulnerability should be viewed as an emergent property of interacting imaging signals, underscoring the limitations of single-marker interpretations in clinical practice [70].

**Fig. 1.**
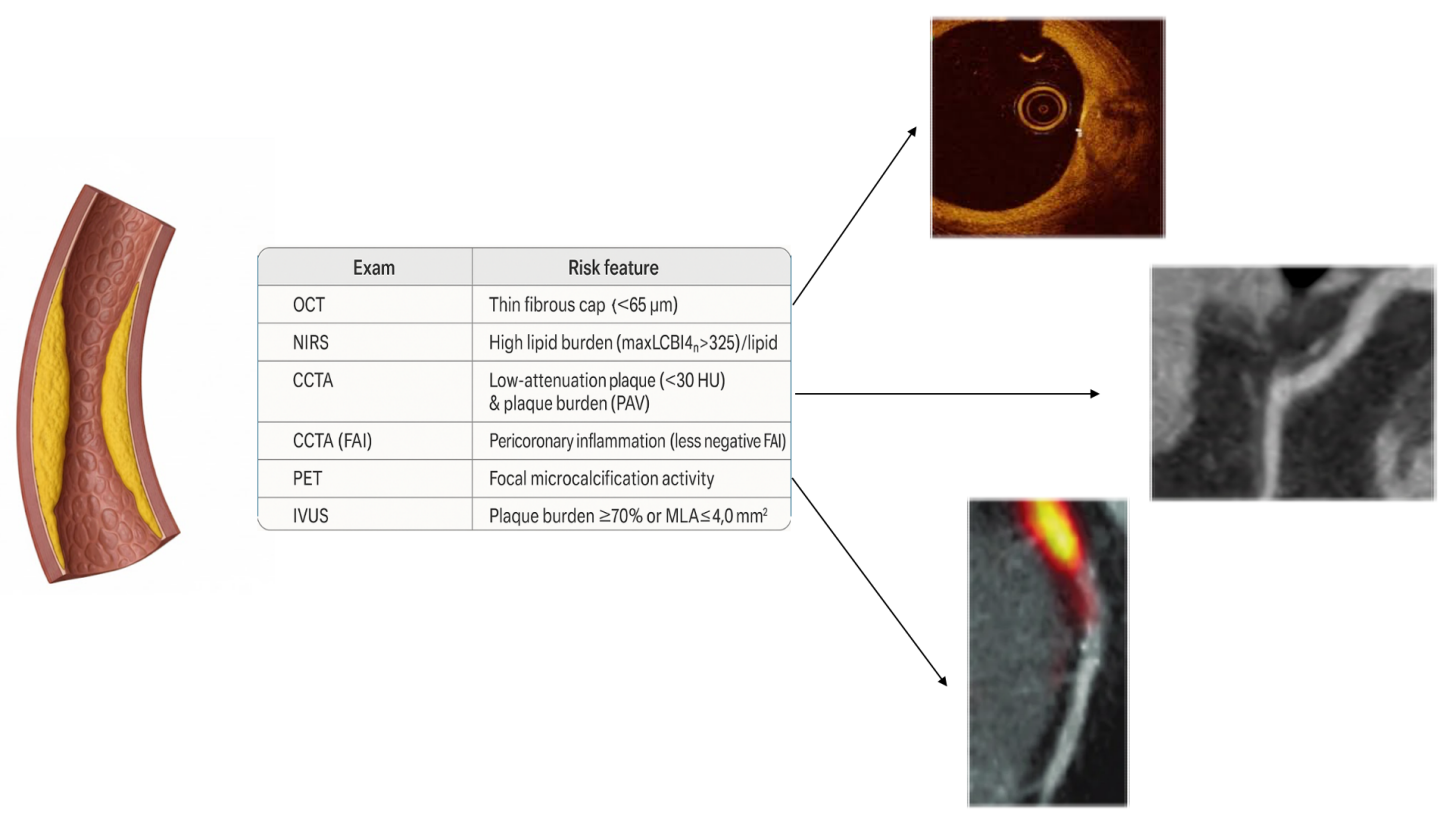
**Coronary imaging modalities and high-risk plaque (HRP) features**. OCT, optical coherence tomography; NIRS, near-infrared spectroscopy; LCBI, lipid core burden index; CCTA, coronary computed tomography angiography; PAV, percent atheroma volume; FAI, fat attenuation index; PET, positron emission tomography; HU, Hounsfield units; IVUS, intravascular ultrasound; MLA, minimal lumen area.

The Fig. 1 shows how multimodality imaging allows a comprehensive identification of HRP.

### 4.2 Risk-Adapted Pathways and Clinical Integration

Risk-adapted care turns multimodal imaging into two levers of action: how intensively we treat and how closely we follow up.

After infarction, adverse biology can worsen before lumen change is obvious—serial intravascular imaging shows rising TCFA prevalence, expanding necrotic core, and only modest minimal lumen area (MLA) change—so lesions with biological deterioration deserve earlier reassessment even when angiographic progression is limited [64, 65]. At baseline, the cumulative weight of vulnerability features should guide prevention intensity across modalities: on OCT, thin cap with macrophage signal identifies higher risk [14, 52]; on NIRS–IVUS, high lipid content within large-burden plaques raises patient-level risk despite modest lesion-level PPV [27, 60]; on CCTA, low-attenuation burden and positive remodelling add risk beyond stenosis, while CT-FFR co-localises anatomy and physiology to prioritise management [33, 34, 40].

A simple triage schema is practical:

(i) Escalate now when multiple high-risk features cluster or worsen on short-interval imaging, prioritising high-intensity lipid lowering, strict blood-pressure targets, smoking cessation, weight and fitness goals, and—in selected post-myocardial infarction patients—evidence-based anti-inflammatory therapy [71, 72, 73, 74]. For lesions with negative fractional flow reserve (FFR >0.80) but imaging-defined high-risk features in non-culprit vessels, PREVENT provides the first randomised signal that preventive percutaneous coronary intervention (PCI) can reduce a 2-year composite endpoint versus optimal medical therapy; however, given the open-label design, relatively short follow-up, and a composite including softer outcomes, routine prophylactic PCI should await independent replication, longer follow-up, and agreed selection criteria—outside trials it should be considered case-by-case in expert centres [29, 34, 53, 75, 76];

(ii) Intensified surveillance when vulnerability signals are present but stable, with earlier revisit if symptoms evolve; choice of modality should reflect the signal to be tracked, e.g., cap on OCT, lipid burden on NIRS–IVUS, low-attenuation volume or FAI on CCTA [53, 77, 78];

(iii) Standard surveillance when high-risk features are absent, and physiology is reassuring, reserving re-imaging for clinical change or risk-factor relapse.

Follow-up should be trajectory-aware rather than schedule-driven: shortening intervals when microstructural risk accumulates despite limited anatomical change, and lengthening them when cap thickens, and inflammatory surrogates abate under optimal therapy [47, 53].

Where available, overlays of endothelial shear and structural-stress maps on CT geometries can flag segments where mechanical fatigue and inflammatory activity co-localise, aligning surveillance and the timing of therapy with the window in which risk is most likely to declare itself [69].

Because the set of imaging features is more informative than any single sign, reports should summarize and integrate both the feature burden and a trajectory class (worsening, stable, improving). This could provide a concise and reproducible report that allows clinicians to use imaging biology information in bedside decisions and support shared decision-making, given the modest positive predictive value at the lesion level in natural history studies.

**Fig. 2.**
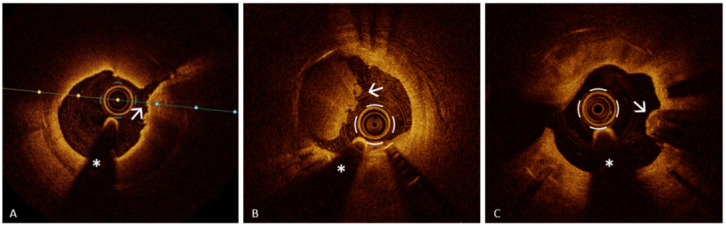
**OCT features of neoatherosclerosis**. Representative OCT examples of neoatherosclerosis showing key morphologic patterns observed in late stent failure. (A) Plaque rupture–like morphology (arrow) with overlying microthrombi in lipid-rich neointima with a thin fibrous cap. (B) Definite OCT erosion with mixed thrombus (arrow) in fibrocalcific neoatherosclerosis. (C) Eruptive calcified nodule with irregular luminal surface (arrow) in diffuse calcific neoatherosclerosis. The asterisk indicates the guidewire artifact. Reproduced from Buonpane A *et al*. [79]. Licensed under CC BY 4.0. OCT, optical coherence tomography; CN, calcified nodule; PR, plaque rupture.

Neoatherosclerosis, a pathologic evolution of neointimal healing, may drive late stent failure and even very late stent thrombosis. OCT typically reveals lipid-rich or calcific neointima, macrophage accumulations, microvessels, or plaque-rupture–like features, as illustrated in Fig. 2 (Ref. [79]).

## 5. Artificial Intelligence and Predictive Integration

AI is redefining cardiovascular imaging by turning plaque evaluation into a predictive and quantitative science [80, 81]. In REVEALPLAQUE, deep-learning analysis of CCTA closely matched IVUS–based references, enabling standardized, serial quantification of total and component plaque from routine scans [82]. Fully automated, end-to-end pipelines likewise classified stenosis and high-risk features with strong agreement and near–real-time turnaround, supporting scalable reporting without manual post-processing [83]. A contemporary systematic review corroborates high concordance and promising risk prediction but highlights heterogeneity and the need for prospective, outcome-linked validation; meanwhile, a transformer model for CT-based plaque erosion showed high diagnostic performance, pointing to non-invasive, potentially stent-sparing phenotyping pending multi-vendor external confirmation [84, 85].

AI-based plaque quantification has emerged as one of the most immediate clinical applications. In the Decisions for Treating Coronary Disease are Changed in Patients Evaluated with Quantified Plaque Analysis (DECODE) study, the integration of automated plaque analysis (AI-QCPA, HeartFlow, Inc., Redwood City, CA, USA) into standard CCTA interpretation led to reclassification of clinical management in two-thirds of patients, primarily by intensifying preventive therapy. This reclassification was most pronounced in cases with higher calcium burden or NCP, demonstrating that quantitative, standardized metrics can expose disease activity overlooked by conventional visual reading [86].

Across CCTA, IVUS, and OCT, AI-enabled pipelines show high concordance with intravascular reference measurements and reduce reader dependence, enabling standardized plaque-burden reporting and supporting efforts to derive population-, age-, and sex-specific reference curves for individualized risk assessment [87, 88].

From a technical standpoint, convolutional neural networks now automate segmentation, plaque composition analysis, and CAD-RADS 2.0 classification, integrating perivascular adipose-tissue mapping as a surrogate marker of coronary inflammation [84]. Machine-learning computation of computed tomography–derived fractional flow reserve (CT-FFR) now couples anatomic and physiologic readouts from the same CCTA scan; in acute chest pain cohorts, it was feasible in ~70% and tracked clinical outcomes for triage, though it adds little when frank plaque rupture is present [89]. In stable/suspected CAD, on-site CT-FFR would substantially cut downstream testing, reclassify management in ~50–60%, and reduce unnecessary invasive coronary angiography—supporting CT-first pathways (CRESCENT I/II). In parallel, radiomic models harvest texture signatures of lipid heterogeneity, microcalcification, and inflammatory tone, delivering a non-invasive “digital phenotype” of vulnerability that complements CT-FFR for risk-adapted care [90].

Longitudinal applications of AI are enabling temporal mapping of disease activity. Dynamic models quantify the velocity of change in fibrous-cap thickness, lipid burden, or perivascular attenuation, turning static imaging into a continuous measure of biological motion. This approach operationalizes the concept of Trajectory-Anchored Quantification, where the rate and direction of biological change—rather than absolute morphology—encode the patient’s evolving risk [69].

The integration of imaging data with biochemical and clinical variables forms the basis for risk-adapted prediction. Federated learning and cloud-based analytics allow multicenter algorithm refinement without sharing raw data, preserving privacy while improving generalizability. AI-driven platforms can thus merge quantitative imaging, inflammatory markers, and clinical context into dynamic models that predict residual risk and therapeutic response in near real time [91, 92].

Within the cath lab, AI for IVOCT now automates lumen/plaque segmentation and component typing, reducing interpretation time and variability and moving IVOCT toward real-time decision support, though broad clinical uptake still requires multicenter validation and workflow integration [93]. In parallel, where adherence to guideline-directed therapy is high, the incremental prognostic yield of “vulnerable plaque” markers over stenosis/burden appears modest—arguing that AI phenotyping should complement, not replace, systematic prevention [78]. Nonetheless, AI-guided quantitative CCTA plaque staging adds long-term prognostic value beyond calcium and stenosis and provides a practical substrate for therapy monitoring and risk tracking [94].

Still, current AI has temporary constraints: heterogeneous plaque definitions and acquisition protocols, small single-vendor datasets, and opaque models limit generalizability and trust—multicenter, outcome-linked validation (e.g., the Oxford Risk Factors And Non-invasive Imaging (ORFAN) study) and standardized ground truths are needed [90]. Bias can also enter at sampling, labeling, and deployment, yielding uneven performance across sex, race, and socioeconomic groups; equity-by-design, transparent reporting, and continuous auditing are essential to mitigate harm [90, 95].

Key areas where AI integrates into the coronary imaging workflow are outlined in Table 2 (Ref. [69, 82, 83, 84, 86, 89, 95]).

**Table 2.** **AI touchpoints in the workflow**.

	Workflow touchpoint	AI capability (examples)	Primary benefit	Reference
1	CCTA plaque segmentation & quantification	REVEALPLAQUE; end-to-end pipelines for stenosis/HRP	Fast, standardized plaque metrics at scale	[82, 83, 84]
2	Physiology from anatomy	ML CT-FFR computed from CCTA	Coupled anatomic-physiologic triage; fewer unnecessary ICA	[89]
3	Longitudinal monitoring & trajectory mapping	Dynamic models (cap thickness, lipid burden, FAI)	Turns snapshots into rates of change for follow-up planning	[69, 95]
4	Management impact & reclassification	Automated AI-QCPA (DECODE)	Up-tiers prevention; reclassifies care	[86]

**Acronym key**. AI, artificial intelligence; ML, machine learning; CCTA, coronary computed tomography angiography; HRP, high-risk plaque; CT-FFR, computed tomography–derived fractional flow reserve; ICA, invasive coronary angiography; FAI, fat attenuation index; DECODE, Decisions for Treating Coronary Disease are Changed in Patients Evaluated with Quantified Plaque Analysis (DECODE) study.

## 6. Conclusion

Atherosclerosis is a dynamic, systemic disease: biology and biomechanics—not stenosis alone—govern risk. Multimodal imaging (IVUS/OCT/NIRS, CCTA/FAI, CMR, PET) converges on the same pathology, and clustered features outperform any single sign. Standardized thresholds and serial, reproducible acquisition convert imaging into a biomarker of change; a concise Biological Activity Index unifying cap integrity, lipid–inflammation (LCBI, FAI, NaF), and mechanical stress can anchor cross-modality reporting. Care should be risk-adapted: escalate when signals cluster or worsen, tighten surveillance when stable, and de-intensify as biology cools; prophylactic PCI remains investigational beyond trials. AI already scales quantification and trajectory mapping, but must be transparent, audited for bias, and validated across vendors and populations.

The paradigm shifts from the vulnerable plaque to the vulnerable patient—and the vulnerable time—enabling personalized prevention that is measurable, repeatable, and clinically actionable.

## References

[1] Global Burden of Cardiovascular Diseases and Risks 2023 Collaborators (2025). Global, Regional, and National Burden of Cardiovascular Diseases and Risk Factors in 204 Countries and Territories, 1990–2023. *Journal of the American College of Cardiology*.

[2] Townsend N, Kazakiewicz D, Lucy Wright F, Timmis A, Huculeci R, Torbica A (2022). Epidemiology of cardiovascular disease in Europe. *Nature Reviews. Cardiology*.

[3] Stone GW, Ali ZA (2023). Detection of Vulnerable Plaque With Intravascular Imaging: Case Closed. *Journal of the American College of Cardiology*.

[4] Weber C, Noels H (2011). Atherosclerosis: current pathogenesis and therapeutic options. *Nature Medicine*.

[5] Ajoolabady A, Pratico D, Lin L, Mantzoros CS, Bahijri S, Tuomilehto J (2024). Inflammation in atherosclerosis: pathophysiology and mechanisms. *Cell Death & Disease*.

[6] Wiyono AV, Ardinal AP, Raharjo PP (2025). Unraveling the significance of innate inflammation in vascular disease. *International Reviews of Immunology*.

[7] Tasouli-Drakou V, Ogurek I, Shaikh T, Ringor M, DiCaro MV, Lei K (2025). Atherosclerosis: A Comprehensive Review of Molecular Factors and Mechanisms. *International Journal of Molecular Sciences*.

[8] Jebari-Benslaiman S, Galicia-García U, Larrea-Sebal A, Olaetxea JR, Alloza I, Vandenbroeck K (2022). Pathophysiology of Atherosclerosis. *International Journal of Molecular Sciences*.

[9] Kardassis D, Vindis C, Stancu CS, Toma L, Gafencu AV, Georgescu A (2025). Unravelling molecular mechanisms in atherosclerosis using cellular models and omics technologies. *Vascular Pharmacology*.

[10] Toth PP, Sniderman AD (2023). Coronary Atherosclerosis: Causes, Consequences, and the Passage of Time. *JACC. Advances*.

[11] Weber C, Habenicht AJR, von Hundelshausen P (2023). Novel mechanisms and therapeutic targets in atherosclerosis: inflammation and beyond. *European Heart Journal*.

[12] Stone PH, Libby P, Boden WE (2023). Fundamental Pathobiology of Coronary Atherosclerosis and Clinical Implications for Chronic Ischemic Heart Disease Management-The Plaque Hypothesis: A Narrative Review. *JAMA Cardiology*.

[13] Gerhardt T, Haghikia A, Stapmanns P, Leistner DM (2022). Immune Mechanisms of Plaque Instability. *Frontiers in Cardiovascular Medicine*.

[14] Lin S, Yu Y, Söderström LÅ, Gisterå A (2024). Erosion of the Atheroma: Wicked T Cells at the Culprit Site. *Current Atherosclerosis Reports*.

[15] Tomaniak M, Katagiri Y, Modolo R, de Silva R, Khamis RY, Bourantas CV (2020). Vulnerable plaques and patients: state-of-the-art. *European Heart Journal*.

[16] Liang M, Puri A, Devlin G (2011). The vulnerable plaque: the real villain in acute coronary syndromes. *The Open Cardiovascular Medicine Journal*.

[17] Libby P, Nahrendorf M, Swirski FK (2016). Leukocytes Link Local and Systemic Inflammation in Ischemic Cardiovascular Disease: An Expanded “Cardiovascular Continuum”. *Journal of the American College of Cardiology*.

[18] Xie Y, Mintz GS, Yang J, Doi H, Iñiguez A, Dangas GD (2014). Clinical outcome of nonculprit plaque ruptures in patients with acute coronary syndrome in the PROSPECT study. *JACC. Cardiovascular Imaging*.

[19] Erlinge D, Maehara A, Ben-Yehuda O, Bøtker HE, Maeng M, Kjøller-Hansen L (2021). Identification of vulnerable plaques and patients by intracoronary near-infrared spectroscopy and ultrasound (PROSPECT II): a prospective natural history study. *Lancet*.

[20] Kang SJ, Mintz GS, Pu J, Sum ST, Madden SP, Burke AP (2015). Combined IVUS and NIRS detection of fibroatheromas: histopathological validation in human coronary arteries. *JACC. Cardiovascular Imaging*.

[21] Legutko J, Bryniarski KL, Kaluza GL, Roleder T, Pociask E, Kedhi E (2022). Intracoronary Imaging of Vulnerable Plaque-From Clinical Research to Everyday Practice. *Journal of Clinical Medicine*.

[22] Chamié D, Wang Z, Bezerra H, Rollins AM, Costa MA (2011). Optical Coherence Tomography and Fibrous Cap Characterization. *Current Cardiovascular Imaging Reports*.

[23] Sinclair H, Bourantas C, Bagnall A, Mintz GS, Kunadian V (2015). OCT for the identification of vulnerable plaque in acute coronary syndrome. *JACC. Cardiovascular Imaging*.

[24] Covani M, Niccoli G, Fujimoto D, Scalamera R, Vergallo R, Porto I (2025). Plaque Vulnerability and Cardiovascular Risk Factor Burden in Acute Coronary Syndrome: An Optical Coherence Tomography Analysis. *Journal of the American College of Cardiology*.

[25] Garcia-Garcia HM, Sanz-Sanchez J, Pinilla-Echeverri N, Blanco PJ, Bourantas C, Alfonso F (2025). Advances in coronary imaging of atherosclerotic plaques. *EuroIntervention*.

[26] Marfella R, Prattichizzo F, Sardu C, Paolisso P, D’Onofrio N, Scisciola L (2023). Evidence of an anti-inflammatory effect of PCSK9 inhibitors within the human atherosclerotic plaque. *Atherosclerosis*.

[27] Kim W, Kook H, Park S, Heo R, Park JK, Shin J (2025). Impact of Post-PCI Lipid Core Burden Index on Angiographic and Clinical Outcomes: Insights From NIRS-IVUS. *Circulation. Cardiovascular Imaging*.

[28] Emfietzoglou M, Mavrogiannis MC, García-García HM, Stamatelopoulos K, Kanakakis I, Papafaklis MI (2023). Current Toolset in Predicting Acute Coronary Thrombotic Events: The “Vulnerable Plaque” in a “Vulnerable Patient” Concept. *Life*.

[29] Ramasamy A, Parasa R, Sokooti H, Zhang X, Tanboga IH, Kitslaar P (2024). Computed tomography versus near-infrared spectroscopy for the assessment of coronary atherosclerosis. *EuroIntervention*.

[30] Aguirre AD, Arbab-Zadeh A, Soeda T, Fuster V, Jang IK (2021). Optical Coherence Tomography of Plaque Vulnerability and Rupture: JACC Focus Seminar Part 1/3. *Journal of the American College of Cardiology*.

[31] Yao Y, Zhang P (2023). Novel ultrasound techniques in the identification of vulnerable plaques-an updated review of the literature. *Frontiers in Cardiovascular Medicine*.

[32] Lu G, Ye W, Ou J, Li X, Tan Z, Li T (2021). Coronary Computed Tomography Angiography Assessment of High-Risk Plaques in Predicting Acute Coronary Syndrome. *Frontiers in Cardiovascular Medicine*.

[33] SCOT-HEART investigators (2015). CT coronary angiography in patients with suspected angina due to coronary heart disease (SCOT-HEART): an open-label, parallel-group, multicentre trial. *Lancet*.

[34] Lee SE, Chang HJ, Rizvi A, Hadamitzky M, Kim YJ, Conte E (2016). Rationale and design of the Progression of AtheRosclerotic PlAque DetermIned by Computed TomoGraphic Angiography IMaging (PARADIGM) registry: A comprehensive exploration of plaque progression and its impact on clinical outcomes from a multicenter serial coronary computed tomographic angiography study. *American Heart Journal*.

[35] Hoffmann U, Truong QA, Schoenfeld DA, Chou ET, Woodard PK, Nagurney JT (2012). Coronary CT angiography versus standard evaluation in acute chest pain. *The New England Journal of Medicine*.

[36] Puchner SB, Liu T, Mayrhofer T, Truong QA, Lee H, Fleg JL (2014). High-risk plaque detected on coronary CT angiography predicts acute coronary syndromes independent of significant stenosis in acute chest pain: results from the ROMICAT-II trial. *Journal of the American College of Cardiology*.

[37] Oikonomou EK, Marwan M, Desai MY, Mancio J, Alashi A, Hutt Centeno E (2018). Non-invasive detection of coronary inflammation using computed tomography and prediction of residual cardiovascular risk (the CRISP CT study): a post-hoc analysis of prospective outcome data. *Lancet*.

[38] Theofilis P, Sagris M, Antonopoulos AS, Oikonomou E, Tsioufis K, Tousoulis D (2022). Non-Invasive Modalities in the Assessment of Vulnerable Coronary Atherosclerotic Plaques. *Tomography*.

[39] Spagnolo M, Giacoppo D, Laudani C, Greco A, Finocchiaro S, Mauro MS (2025). Advances in the Detection and Management of Vulnerable Coronary Plaques. *Circulation. Cardiovascular Interventions*.

[40] Lorenzatti D, Piña P, Huang D, Apple SJ, Fernandez-Hazim C, Ippolito P (2024). Interaction between risk factors, coronary calcium, and CCTA plaque characteristics in patients aged 18-45 years. *European Heart Journal. Cardiovascular Imaging*.

[41] Yang Y, Shah JP, Zeng H, Yu J, Hagar A, Sihag V (2020). Prevalence and Prognosis of High-Risk Plaque on Coronary CT Angiography in Hospitalized Patients. *JACC. Cardiovascular Imaging*.

[42] Wang X, van den Hoogen IJ, Butcher SC, Kuneman JH, de Graaf MA, Kamperidis V (2023). Importance of plaque volume and composition for the prediction of myocardial ischaemia using sequential coronary computed tomography angiography/positron emission tomography imaging. *European Heart Journal. Cardiovascular Imaging*.

[43] Fukase T, Dohi T, Fujimoto S, Nishio R, Nozaki YO, Kudo A (2023). Relationship between coronary high-intensity plaques on T1-weighted imaging by cardiovascular magnetic resonance and vulnerable plaque features by near-infrared spectroscopy and intravascular ultrasound: a prospective cohort study. *Journal of Cardiovascular Magnetic Resonance*.

[44] Isodono K, Matsumoto H, Li D, Slomka PJ, Dey D, Cadet S (2024). Coronary Plaque Characterization with T1-weighted MRI and Near-Infrared Spectroscopy to Predict Periprocedural Myocardial Injury. *Radiology. Cardiothoracic Imaging*.

[45] Coolen BF, Calcagno C, van Ooij P, Fayad ZA, Strijkers GJ, Nederveen AJ (2018). Vessel wall characterization using quantitative MRI: what’s in a number?. *MAGMA*.

[46] Currie G, Kiat H (2025). Beyond the Lumen: Molecular Imaging to Unmask Vulnerable Coronary Plaques. *Journal of Cardiovascular Development and Disease*.

[47] Vergallo R, Park SJ, Stone GW, Erlinge D, Porto I, Waksman R (2025). Vulnerable or High-Risk Plaque: A JACC: Cardiovascular Imaging Position Statement. *JACC. Cardiovascular Imaging*.

[48] Suzuki K, Kinoshita D, Yuki H, Niida T, Sugiyama T, Yonetsu T (2024). Higher Noncalcified Plaque Volume Is Associated With Increased Plaque Vulnerability and Vascular Inflammation. *Circulation. Cardiovascular Imaging*.

[49] van Veelen A, van der Sangen NMR, Delewi R, Beijk MAM, Henriques JPS, Claessen BEPM (2022). Detection of Vulnerable Coronary Plaques Using Invasive and Non-Invasive Imaging Modalities. *Journal of Clinical Medicine*.

[50] Achenbach S (2020). Imaging the Vulnerable Plaque on Coronary CTA. *JACC. Cardiovascular Imaging*.

[51] Prati F, Arbustini E, Alfonso F (2022). Skating on thin ice: searching for vulnerable plaques. *EuroIntervention*.

[52] Tufaro V, Jaffer FA, Serruys PW, Onuma Y, van der Steen AFW, Stone GW (2024). Emerging Hybrid Intracoronary Imaging Technologies and Their Applications in Clinical Practice and Research. *JACC. Cardiovascular Interventions*.

[53] Rigattieri S, Redivo M, Casenghi M, Belmonte M, Giovannelli F, Tommasino A (2025). Management of Coronary Vulnerable Plaques: A Focus on Preventive Percutaneous Coronary Intervention. *Reviews in Cardiovascular Medicine*.

[54] Zhang Y, Taylor E, Huang N, Hamilton J, Cheng JX (2022). Survival intravascular photoacoustic imaging of lipid-rich plaque in cholesterol fed rabbits. *Translational Biophotonics*.

[55] Yuan Y, Zhang G, Chen Y, Ni H, Li M, Sturek M (2023). A high-sensitivity high-resolution intravascular photoacoustic catheter through mode cleaning in a graded-index fiber. *Photoacoustics*.

[56] Figtree GA, Adamson PD, Antoniades C, Blumenthal RS, Blaha M, Budoff M (2022). Noninvasive Plaque Imaging to Accelerate Coronary Artery Disease Drug Development. *Circulation*.

[57] Goeller M, Tamarappoo BK, Kwan AC, Cadet S, Commandeur F, Razipour A (2019). Relationship between changes in pericoronary adipose tissue attenuation and coronary plaque burden quantified from coronary computed tomography angiography. *European Heart Journal. Cardiovascular Imaging*.

[58] Nurmohamed NS, van Rosendael AR, Danad I, Ngo-Metzger Q, Taub PR, Ray KK (2024). Atherosclerosis evaluation and cardiovascular risk estimation using coronary computed tomography angiography. *European Heart Journal*.

[59] Dweck MR, Fayad ZA (2017). Multitarget Vulnerable Plaque Imaging. *Circulation. Cardiovascular Imaging*.

[60] Roleder T, Kovacic JC, Ali Z, Sharma R, Cristea E, Moreno P (2014). Combined NIRS and IVUS imaging detects vulnerable plaque using a single catheter system: a head-to-head comparison with OCT. *EuroIntervention*.

[61] Min JK, Chang HJ, Andreini D, Pontone G, Guglielmo M, Bax JJ (2022). Coronary CTA plaque volume severity stages according to invasive coronary angiography and FFR. *Journal of Cardiovascular Computed Tomography*.

[62] Gallone G, Bellettini M, Gatti M, Tore D, Bruno F, Scudeler L (2023). Coronary Plaque Characteristics Associated With Major Adverse Cardiovascular Events in Atherosclerotic Patients and Lesions: A Systematic Review and Meta-Analysis. *JACC. Cardiovascular Imaging*.

[63] Bourantas CV, Papafaklis MI, Athanasiou L, Kalatzis FG, Naka KK, Siogkas PK (2013). A new methodology for accurate 3-dimensional coronary artery reconstruction using routine intravascular ultrasound and angiographic data: implications for widespread assessment of endothelial shear stress in humans. *EuroIntervention*.

[64] Zhao Z, Witzenbichler B, Mintz GS, Jaster M, Choi SY, Wu X (2013). Dynamic nature of nonculprit coronary artery lesion morphology in STEMI: a serial IVUS analysis from the HORIZONS-AMI trial. *JACC. Cardiovascular Imaging*.

[65] Tian J, Dauerman H, Toma C, Samady H, Itoh T, Kuramitsu S (2014). Prevalence and characteristics of TCFA and degree of coronary artery stenosis: an OCT, IVUS, and angiographic study. *Journal of the American College of Cardiology*.

[66] Sgreva S, Alsubai SE, Bianchini E, Alqahtani F, Del Sole PA, Elzomor H (2025). Plaques Do Not Act Alone: Time to Redefine Coronary Vulnerability from Lesion to Phenotype. *Journal of Clinical Medicine*.

[67] Tang D, Yang C, Huang S, Mani V, Zheng J, Woodard PK (2017). Cap inflammation leads to higher plaque cap strain and lower cap stress: An MRI-PET/CT-based FSI modeling approach. *Journal of Biomechanics*.

[68] Moss A, Daghem M, Tzolos E, Meah MN, Wang KL, Bularga A (2023). Coronary Atherosclerotic Plaque Activity and Future Coronary Events. *JAMA Cardiology*.

[69] Schulze K, Stantien AM, Williams MC, Vassiliou VS, Giannopoulos AA, Nieman K (2026). Coronary CT angiography evaluation with artificial intelligence for individualized medical treatment of atherosclerosis: a Consensus Statement from the QCI Study Group. *Nature Reviews. Cardiology*.

[70] Kumar P, Bhatia M (2022). Coronary Artery Disease Reporting and Data System: A Comprehensive Review. *Journal of Cardiovascular Imaging*.

[71] Aldana-Bitar J, Golub IS, Moore J, Krishnan S, Verghese D, Manubolu VS (2024). Colchicine and plaque: A focus on atherosclerosis imaging. *Progress in Cardiovascular Diseases*.

[72] Zanda G, Varbella F (2023). Stabilization of vulnerable plaque in the ACS patient: evidence from HUYGENS studies. *European Heart Journal Supplements*.

[73] Barbieri L, Tumminello G, Fichtner I, Corsini A, Santos RD, Carugo S (2024). PCSK9 and Coronary Artery Plaque-New Opportunity or Red Herring?. *Current Atherosclerosis Reports*.

[74] Dawson LP, Lum M, Nerleker N, Nicholls SJ, Layland J (2022). Coronary Atherosclerotic Plaque Regression: JACC State-of-the-Art Review. *Journal of the American College of Cardiology*.

[75] Park SJ, Ahn JM, Kang DY, Yun SC, Ahn YK, Kim WJ (2024). Preventive percutaneous coronary intervention versus optimal medical therapy alone for the treatment of vulnerable atherosclerotic coronary plaques (PREVENT): a multicentre, open-label, randomised controlled trial. *Lancet*.

[76] Park DW, Kim H, Singh A, Brown DL (2024). Prophylactic stenting of vulnerable plaques: pros and cons. *EuroIntervention*.

[77] Khialani B, Alfonso F, Malakouti S, Rodrigues ACLF, Reddy K, Garcia HM (2025). Preventive Percutaneous Intervention of Vulnerable Coronary Plaques. *The American Journal of Cardiology*.

[78] Bairey Merz CN (2024). Vulnerable plaque and major adverse cardiovascular events: anatomy of a failure. *European Heart Journal*.

[79] Buonpane A, Trimarchi G, Ciardetti M, Coceani MA, Alagna G, Benedetti G (2024). Optical Coherence Tomography in Myocardial Infarction Management: Enhancing Precision in Percutaneous Coronary Intervention. *Journal of Clinical Medicine*.

[80] Veneziano FA, Gioia FA, Gentile F (2025). Hybrid PET/CT and PET/MR in Coronary Artery Disease: An Update for Clinicians, with Insights into AI-Guided Integration. *Journal of Cardiovascular Development and Disease*.

[81] Föllmer B, Williams MC, Dey D, Arbab-Zadeh A, Maurovich-Horvat P, Volleberg RHJA (2024). Roadmap on the use of artificial intelligence for imaging of vulnerable atherosclerotic plaque in coronary arteries. *Nature Reviews. Cardiology*.

[82] Narula J, Stuckey TD, Nakazawa G, Ahmadi A, Matsumura M, Petersen K (2024). Prospective deep learning-based quantitative assessment of coronary plaque by computed tomography angiography compared with intravascular ultrasound: the REVEALPLAQUE study. *European Heart Journal. Cardiovascular Imaging*.

[83] Shrivastava P, Kashikar S, Parihar PH, Kasat P, Bhangale P, Shrivastava P (2025). A systematic review on deep learning-enabled coronary CT angiography for plaque and stenosis quantification and cardiac risk prediction. *European Journal of Radiology Open*.

[84] Ihdayhid AR, Sehly A, He A, Joyner J, Flack J, Konstantopoulos J (2024). Coronary Artery Stenosis and High-Risk Plaque Assessed With an Unsupervised Fully Automated Deep Learning Technique. *JACC. Advances*.

[85] Park S, Yuki H, Niida T, Suzuki K, Kinoshita D, McNulty I (2023). A novel deep learning model for a computed tomography diagnosis of coronary plaque erosion. *Scientific Reports*.

[86] Rinehart S, Raible SJ, Ng N, Mullen S, Huey W, Rogers C (2024). Utility of Artificial Intelligence Plaque Quantification: Results of the DECODE Study. *Journal of the Society for Cardiovascular Angiography & Interventions*.

[87] Shin D, Sami Z, Cannata M, Ciftcikal Y, Caron E, Thomas SV (2025). Artificial Intelligence in Intravascular Imaging for Percutaneous Coronary Interventions: A New Era of Precision. *Journal of the Society for Cardiovascular Angiography & Interventions*.

[88] Lauretti A, Borgi M, Versaci F (2025). Artificial intelligence in coronary plaque characterization and risk assessment: from images to impact. *Vessel Plus*.

[89] Eberhard M, Nadarevic T, Cousin A, von Spiczak J, Hinzpeter R, Euler A (2020). Machine learning-based CT fractional flow reserve assessment in acute chest pain: first experience. *Cardiovascular Diagnosis and Therapy*.

[90] Nous FMA, Budde RPJ, Lubbers MM, Yamasaki Y, Kardys I, Bruning TA (2020). Impact of machine-learning CT-derived fractional flow reserve for the diagnosis and management of coronary artery disease in the randomized CRESCENT trials. *European Radiology*.

[91] Klüner LV, Chan K, Antoniades C (2024). Using artificial intelligence to study atherosclerosis from computed tomography imaging: A state-of-the-art review of the current literature. *Atherosclerosis*.

[92] Park S, Araki M, Nakajima A, Lee H, Fuster V, Ye JC (2022). Enhanced Diagnosis of Plaque Erosion by Deep Learning in Patients With Acute Coronary Syndromes. *JACC. Cardiovascular Interventions*.

[93] Sperti M, Cardaci C, Bruno F, Shah STH, Panagiotopoulos K, Kassem K (2025). Artificial Intelligence-based Approaches for Characterizing Plaque Components From Intravascular Optical Coherence Tomography Imaging: Integration Into Clinical Decision Support Systems. *Reviews in Cardiovascular Medicine*.

[94] Nurmohamed NS, Bom MJ, Jukema RA, de Groot RJ, Driessen RS, van Diemen PA (2024). AI-Guided Quantitative Plaque Staging Predicts Long-Term Cardiovascular Outcomes in Patients at Risk for Atherosclerotic CVD. *JACC. Cardiovascular Imaging*.

[95] Mihan A, Pandey A, Van Spall HG (2024). Mitigating the risk of artificial intelligence bias in cardiovascular care. *The Lancet. Digital Health*.

